# CiRS-7 promotes growth and metastasis of esophageal squamous cell carcinoma via regulation of miR-7/HOXB13

**DOI:** 10.1038/s41419-018-0852-y

**Published:** 2018-08-06

**Authors:** Rui-chao Li, Shun Ke, Fan-kai Meng, Jun Lu, Xiao-jing Zou, Zhi-gang He, Weng-feng Wang, Ming-hao Fang

**Affiliations:** 1 Department of General Medicine, Tongji Medical College, Huazhong University of Science & Technology, Wuhan, 430030 China; 2 Department of Emergency Medicine, Tongji Medical College, Huazhong University of Science & Technology, Wuhan, 430030 China; 3 Department of hematology, Tongji Medical College, Huazhong University of Science & Technology, Wuhan, 430030 China; 4 Department of Cardiovascular Medicine, Ezhou Central Hospital of Hubei, Ezhou, 436000 China

## Abstract

The circular RNA ciRS-7 has been reported to be involved in the pathogenesis of various tumors, including gastric and colorectal cancer. However, the role of ciRS-7 in esophageal squamous cell carcinoma (ESCC) remains unsolved. In this study, we found that the ciRS-7 expression was significantly upregulated in ESCC cancer tissues compared with matched normal tissues and associated with poor patient survival. Overexpression of ciRS-7 abrogated the tumor-suppressive roles of miR-7 including cell proliferation, migration and invasion in vitro as well as tumor growth and lung metastasis in vivo. Mechanistically, ciRS-7 functioned as the sponge of miR-7 and reactivated its downstream HOXB13-mediated NF-κB/p65 pathway. Conclusively, our findings demonstrate how ciRS-7 induces malignant progression of ESCC and that ciRS-7 may act as a novel prognostic marker and therapeutic target for this lethal disease.

## Introduction

Esophageal carcinoma is one of the most common gastrointestinal malignancies and the sixth leading cause of cancer-related death worldwide^1,2^. Esophageal carcinoma can be histopathologically divided into two common types: esophageal adenocarcinoma (EAC) and esophageal squamous cell carcinoma (ESCC). Although EAC is increasing rapidly in Western countries, most patients in East Asian especially in China are diagnosed as ESCC^3–5^. The primary issue derived from ESCC is its extremely poor 5-year survival rate due to rare diagnosis made prior to advanced disease stages^1,3^. Curative surgery, which is recommended for early stage cases, is only feasible for 30–40% advanced patients^6,7^. Therefore, there is a compelling need to identify novel biomarkers that can predict ESCC prognosis reliably and to uncover the molecular mechanisms underlying ESCC progression.

Genomic studies have revealed that more than 90% in the human transcripts including microRNA (miRNA), long non-coding RNA (lncRNA), and circular RNA (circRNA) are with limited protein-coding capacity^8–10^. The circRNA is characterized by a covalently closed continuous loop and evolutionarily conservation in various organisms^10,11^. Interestingly, different RNA transcripts that share the same miRNA-binding sites can communicate with and regulate each other by competing for the common sequences of miRNA molecules, which is known as the competing endogenous RNAs (ceRNA) mechanism^12,13^. Accumulating evidence is now unveiling that dysregulation of circRNA plays vital roles in human cancer pathogenesis^14,15^. Recently, a circRNA discovered in human named Cdr1as (antisense to the cerebellar degeneration-related protein 1 transcript), also termed as ciRS-7 (circular RNA sponge for miR-7), was proved to act as a ceRNA of miR-7^16-19^. Moreover, it was reported that soak up of miR-7 by ciRS-7 resulted in release and reactivation of genes previously repressed by miR-7 during the development of brain^18^. Although several studies have revealed downregulation of miR-7 in human disorders and associations with dysregulated key pathways^20–23^, roles of ciRS-7 received less attention. For example, sponge of miR-7 by ciRS-7 abrogated its tumor-suppressive roles in gastric^24^ and colorectal cancer^25^. In ESCC, overexpression of miR-7 significantly inhibits proliferation of tumor cells^23,26^. However, no studies have explored the connections among ciRS-7, miR-7, and clinical features in ESCC.

In the present study, we have attempted for the first time to fill this gap with knowledge relating to the underlying molecular mechanisms of ciRS-7 in ESCC. We specifically set out to investigate its relevance as a prognostic biomarker by profiling the expression level of ciRS-7 in neoplastic tissues and matched normal tissues. In addition, we found that overexpression of ciRS-7 abrogated the tumor-suppressive roles of miR-7 and reactivated the HOXB13-mediated NF-κB/p65 pathway in ESCC through a systematic and comprehensive functional analysis followed by a series of tumor xenograft animal assays. Our results indicated that upregulation of ciRS-7 may serve as a biomarker for prognosis predication and as a potential therapeutic target for ESCC patients.

## Results

### Upregulation of ciRS-7 and downregulation of miR-7 predicts poor prognosis of ESCC patients

**Fig. 1:**
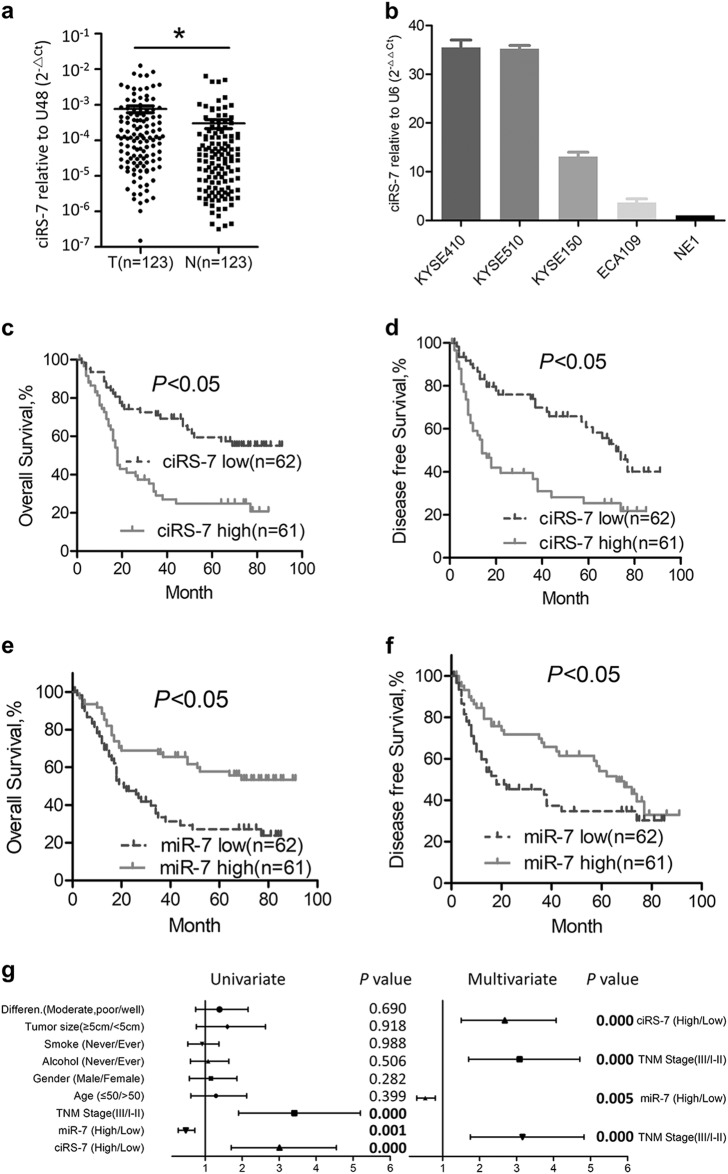
Overexpression of ciRS-7 predicts poor prognosis of ESCC patients. **a** The expression levels of ciRS-7 in ESCC tissues and corresponding non-cancerous tissues was detected by qPCR. **b** Expression of ciRS-7 in a panel of ESCC cancer cell lines and immortalized squamous epithelial cells (NE1). The expression levels of ciRS-7 in ESCC cancer tissues was normalized to that of corresponding non-cancerous tissues. Data was presented as fold change of △Ct. The patients were assigned to high expression group and low expression group using the median fold change as cutoff value. Kaplan–Meier analysis of overall survival (**c**) and disease-free survival (**d**) in ESCC patients with low and high ciRS-7 levels. Kaplan–Meier analysis of overall survival (**e**) and disease-free survival (**f**) in ESCC patients with low and high miR-7 levels. **g** Forest map for univariate and multivariate analysis of overall survival based on ciRS-7 or miR-7 expression level. Data in **b** represents the mean ± SD of three repeated experiments

Oncogenic roles of ciRS-7 has been reported in gastrointestinal^24,25^ and lung cancer^27^. We first evaluated expression level of ciRS-7 in ESCC and found that ciRS-7 was overexpressed in cancer tissues compared with corresponding non-cancerous tissues (Fig. 1a). Moreover, upregulation of ciRS-7 was observed in a panel of ESCC cells in comparison with that in the immortalized NE1 cells (Fig. 1b). The enrolled patients were divided into high expression group (*n* = 61) and low expression group (*n* = 62) using the median level (ciRS-7 or miR-7) as the cutoff value. Overexpression of ciRS-7 was correlated positively with advanced TNM stage and exhibited no significant associations with other parameters including age, gender, alcohol consumption, smoking history, tumor size, or differentiation status (Table 1), while reduced expression of miR-7 correlated significantly with older age in ESCC patient (Table S1). Kaplan–Meier analysis also manifested that ESCC subjects with highly expressed ciRS-7 or lowly expressed miR-7 possessed inferior overall survival (OS) and disease-free survival (DFS) to those with lowly expressed ciRS-7 or highly expressed miR-7, respectively (Fig. 1c–f). In multivariate analysis, ciRS-7, miR-7 expression, and TNM stage were identified as independent prognostic factors for the OS in ESCC (Fig. 1g).

**Table 1:** The correlation between clinicopathological parameters and ciRS-7 expression

	ciRS-7 expression		*P*
	Low, *n* (%)	High, *n* (%)	
Age			
≤50	15(65.2)	8(34.8)	0.165
>50	47(47.0)	53(53.0)	
Gender			
Male	49(49.5)	50(50.5)	0.821
Female	13(54.2)	11(45.8)	
Alcohol consumption			
Ever and current	40(48.2)	43(51.8)	0.565
Never	22(55.0)	18(45.0)	
Smoking status			
Ever and current	24(43.6)	31(56.4)	0.206
Never	38(55.9)	30(44.1)	
Tumor size			
<5 cm	52(50.5)	51(49.5)	1.000
≥5 cm	10(50.0)	10(50.0)	
Differentiation status			
Well or Moderate	49(51.6)	46(48.4)	0.672
Poor	13(46.4)	15(53.6)	
TNM stage			0.048*
I–II	37(59.7)	25(40.3)	
III	25(41.0)	36(59.0)	

### Overexpression of ciRS-7 abrogates the anti-proliferative roles of miR-7 in ESCC

**Fig. 2:**
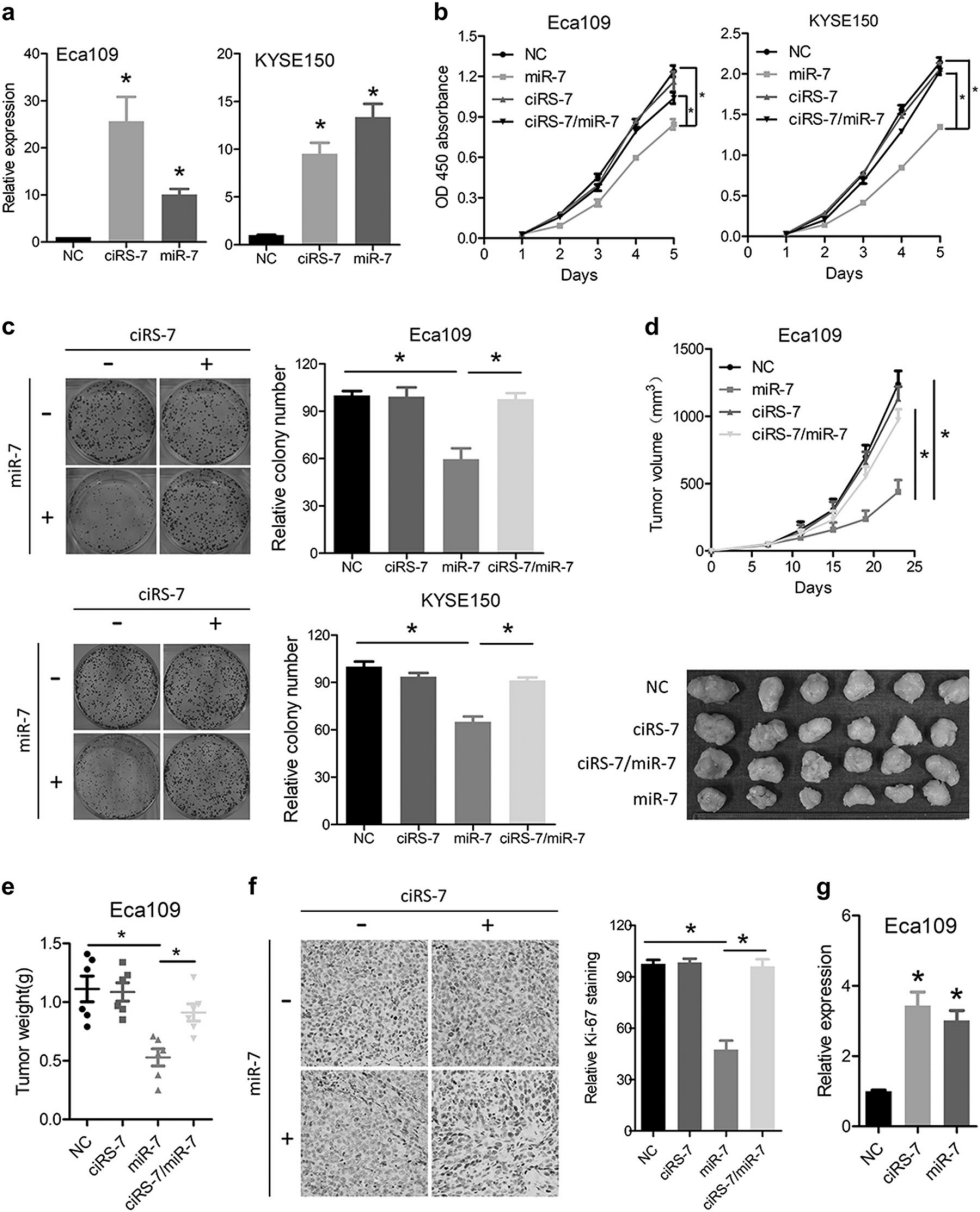
Overexpression of ciRS-7 abrogates the anti-proliferative roles of miR-7 in ESCC. **a** The expression levels of ciRS-7 or miR-7 in Eca109 and KYSE150 cells after manipulative overexpression was detected by qPCR. **b** CCK-8 assays of Eca109 and KYSE150 cells after overexpression of ciRS-7, miR-7, or both. **c** Colony formation assays of Eca109 and KYSE150 cells after overexpression of ciRS-7, miR-7, or both. Left panel was representative images and right panel was statistical quantification. **d** Tumor growth curve and dissected xenografts of Eca109 cells after overexpression of ciRS-7, miR-7, or both. **e** Weight of dissected tumors was recorded. **f** Immunohistochemical analysis of Ki-67 in the dissected tumors. Left panel was representative images and right panel was statistical quantification. **g** Expression level of ciRS-7 or miR-7 in the dissected tumors was detected by qPCR. Data in **a**, **b**, and **c** represents the mean ± SD of three repeated experiments. Data in **d**, **e**, **f**, and **g** represents the mean ± SD of six mice. *P < 0.05

We then selected Eca109 and KYSE150 cells with relative low ciRS-7 expression for further analysis and constructed stable cell lines with ectopically overexpressed miR-7, ciRS-7 or both. quantitative polymerase chain reaction (qPCR) analysis confirmed successful overexpression of ciRS-7 and miR-7 in Eca109 and KYSE150 cells (Fig. 2a). Overexpression of miR-7-inhibited cell growth, which could be abrogated by simultaneous upregulation of ciRS-7 (Fig. 2b). Further assays also indicated that ciRS-7 could interrupt the anti-proliferation ability of miR-7 in Eca109 and KYSE150 cells (Fig. 2c). The indicated cells were implanted subcutaneously into the nude mice and the tumor volumes were monitored. The growth curve indicated that tumors in mice injected with miR-7 overexpressing cells have lower growth rate compared with those injected with ciRS-7 overexpressing or miR-7/ciRS-7 double overexpressing cells (Fig. 2d). Also, the average weight of miR-7 expressing tumors at the time of sacrifice was approximately half that of the other three groups. (Fig. 2e). Immunohistochemical (IHC) staining of Ki-67 showed similar proliferative capacity in the NC, ciRS-7, and ciRS-7/miR-7 group and significant lower cell growth index after overexpression of miR-7 (Fig. 2f). qPCR analysis of the xenografts showed efficient overexpression of ciRS-7 and miR-7 (Fig. 2g).

### Overexpression of ciRS-7 promotes metastasis of ESCC cells via miR-7

**Fig. 3:**
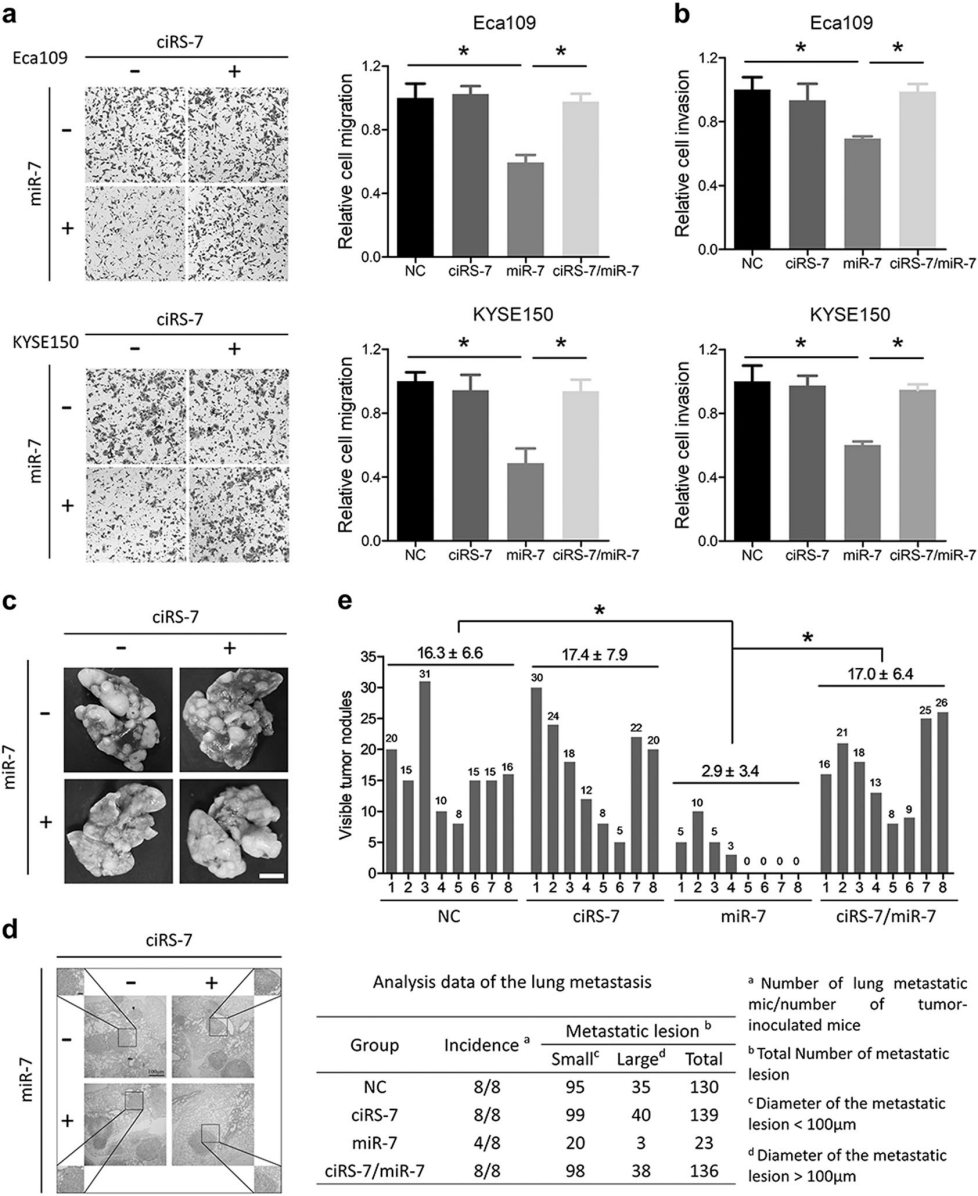
Overexpression of ciRS-7 promotes metastasis of ESCC cells via miR-7 regulation. **a** Migration assays of Eca109 and KYSE150 cells after overexpression of ciRS-7, miR-7, or both. Left panel was representative images and right panel was statistical quantification. **b** Quantification of cell invasion in Eca109 and KYSE150 cells. **c** Lung metastasis of Eca109 cells after tail vein injection. Scale bars: 1 cm. **d** Representative metastatic lesions stained by H&E in the lungs of mice 6 weeks after tail vein injection of indicated cells. Scale bars: 100 μm. **e** Quantitative analysis of number of lung metastatic colonies in each group. Data in **a** and **b** represents the mean ± SD of three repeated experiments. Data in **e** represents the mean ± SD of eight mice. *P < 0.05

We further explored the roles of ciRS-7 overexpression in ESCC via transwell assays. As shown in Fig. 3a, b, overexpression of miR-7 significantly inhibited the migration and invasion ability of Eca109 and KYSE150 cells, and such suppressive effects was neutralized by ectopic ciRS-7 introduction. The Eca109 cells with stably expressing ciRS-7, miR-7, or both were injected into the nude mice via tail vein. Number of the lung metastasis was significantly less in the miR-7 overexpressing group compared with the NC, ciRS-7 overexpressing or ciRS-7/miR-7 double overexpressing group, which showed consistent trend with the in vitro assays (Fig. 3c–e).

### The ciRS-7/miR-7 axis regulates HOXB13 expression in ESCC cells

**Fig. 4:**
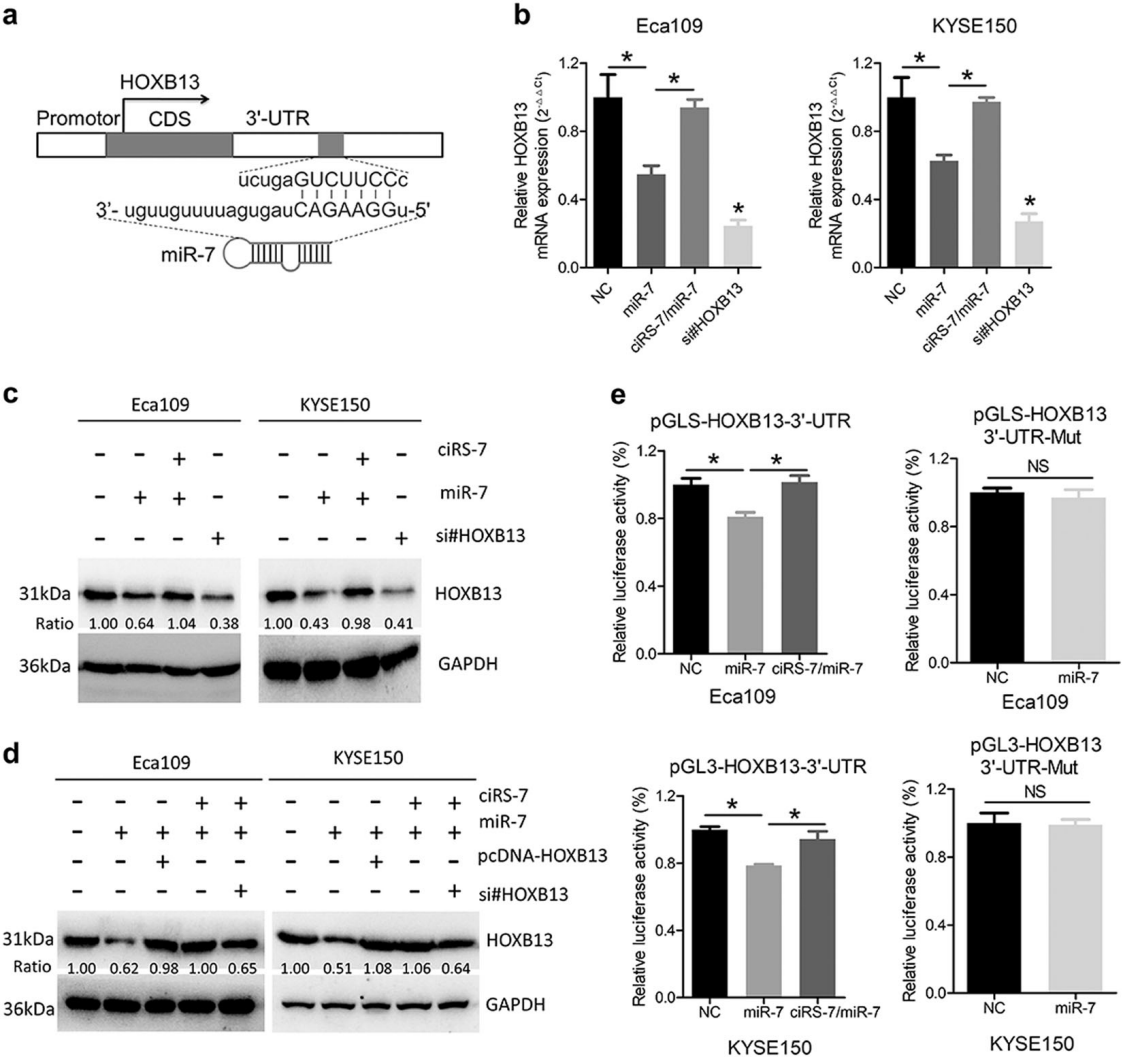
The ciRS-7/miR-7 axis regulates expression of HOXB13 in ESCC cells. **a** Schematic model of interaction between miR-7 and HOXB13. **b** The expression levels of HOXB13 in Eca109 and KYSE150 cells after ciRS-7 or miR-7 overexpression or siRNA transfection was detected by qPCR. **c** Western blot analysis of HOXB13 after ciRS-7 or miR-7 overexpression or siRNA transfection. **d** Western blot analysis of HOXB13 after ciRS-7, miR-7, or HOXB13 overexpression or siRNA transfection. **e** Dual luciferase analysis in Eca109 and KYSE150 cells after overexpression of ciRS-7 or miR-7. Data in b and e represents the mean ± SD of three repeated experiments. *P < 0.05, NS non-significant

Since our data showed that ciRS-7 effectively quenched normal function of miR-7 to suppress ESCC tumorigenesis and metastasis, we hypothesized that ciRS-7 may be responsible for enhancing the expression levels of miR-7 targets by acting as a miR-7 sponge and facilitating a more aggressive phenotype in ESCC patients. HOXB13 was predicted to act as the downstream target of miR-7^28^ through complementary binding sequence in the 3′-UTR (3′-untranslated region) (Fig. 4a) and promotes tumor cell proliferation and metastasis in several human cancers^23,29,30^. Interestingly, we noted a significant downregulation of HOXB13 after overexpression of miR-7 at both the mRNA and protein level, and this decrease could be reversed by ciRS-7 reintroduction (Fig. 4b, c). We constructed a vector expressing the ORF (open reading frame) of HOXB13 without the 3′-UTR, which was designated as pcDNA-HOXB13. Transfection with pcDNA-HOXB13 abrogated the suppressive roles of miR-7 on HOXB13 expression (Fig. 4d). Dual luciferase assays further supported that miR-7 could bind the 3′-UTR of HOXB13 (Fig. 4e).

**Fig. 5:**
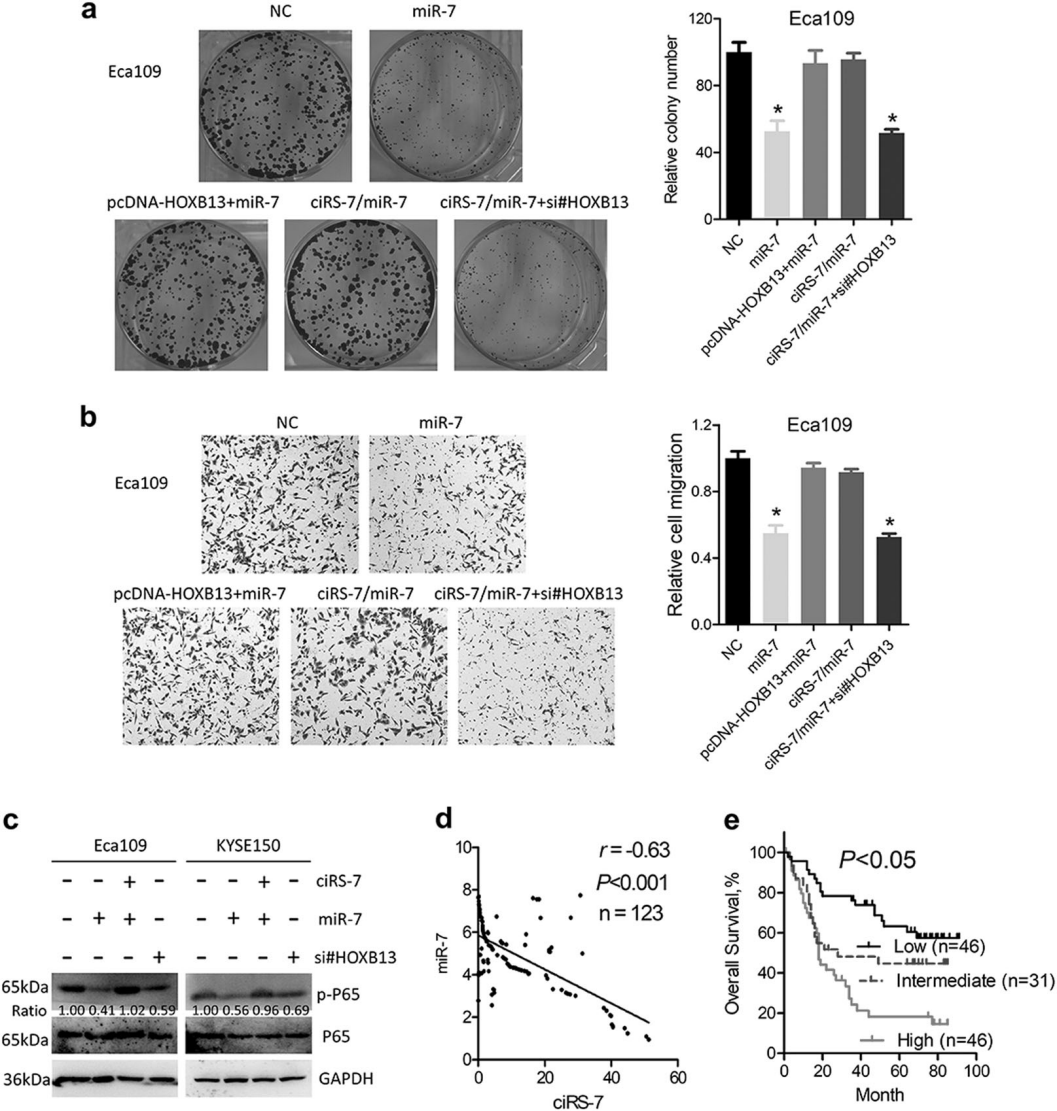
The ciRS-7/miR-7 promotes proliferation and migration of ESCC cells via HOXB13. **a** Colony formation assays of Eca109 cells after overexpression of ciRS-7, miR-7, HOXB13, or siRNA transfection. Left panel was representative images and right panel was statistical quantification. **b** Migration assays of Eca109 cells after overexpression of ciRS-7, miR-7, HOXB13, or siRNA transfection. Left panel was representative images and right panel was statistical quantification. **c** Western blot analysis of phosphor-p65 after ciRS-7 or miR-7 overexpression or siRNA transfection. **d** Correlation between expression level of ciRS-7 and miR-7 in ESCC tumor tissues. **e** Overall survival of ESCC patients based on expression level of ciRS-7 and miR-7 (low risk, low ciRS-7, and high miR-7; intermediate risk, low ciRS-7 and low miR-7, or high ciRS-7 and high miR-7; high risk, high ciRS-,7 and low ciRS-7). Data in **a** and **b** represents the mean ± SD of three repeated experiments. *P < 0.05

To further explore whether ciRS-7/miR-7 promotes malignant phenotype of ESCC via HOXB13, colony formation and migration assays were performed. Cell growth and migration was decreased after miR-7 expression, which could be attenuated by overexpression of HOXB13 (Fig. 5a, b and Supplementary Figure S1A and S1B). Knockdown of HOXB13 in ciRS-7/miR-7 overexpressing cells inhibited colony formation and migration in Eca109 and KYSE150 cells (Fig. 5a, b and Supplementary Figure S1A and S1B).

### Aberrantly activated ciRS-7/miR-7 axis regulates p65 phosphorylation and indicates poor prognosis of ESCC patients

Activation of NF-κB pathway plays vital roles in human diseases including cancer^31,32^ and was reported to be involved in the pro-metastasis effects of HOXB13 in prostate cancer^33^ and to mediate the oncogenic characteristics of ciRS-7 in lung cancer^27^. We therefore hypothesized whether NF-κB pathway was activated by the ciRS-7/miR-7/HOXB13 axis in ESCC. Western blot analysis demonstrated that phosphorylation of p65 mirrored expression of HOXB13, which was regulated by ciRS-7/miR-7 axis in Eca109 and KYSE150 cells (Fig. 5c), providing possible explanation for the oncogenic roles of ciRS-7 in ESCC. The expression of ciRS-7 and miR-7 showed significant negative correlation in ESCC patients (Fig. 5d). When patients were divided into high risk (high ciRS-7 and low miR-7), intermediate risk (high ciRS-7 and high miR-7 or low ciRS-7 and low miR-7), and low risk (low ciRS-7 and high miR-7) group, the overall survival and disease-free survival showed significant difference in these three groups (Fig. 5e and Supplementary Figure S2).

**Fig. 6:**
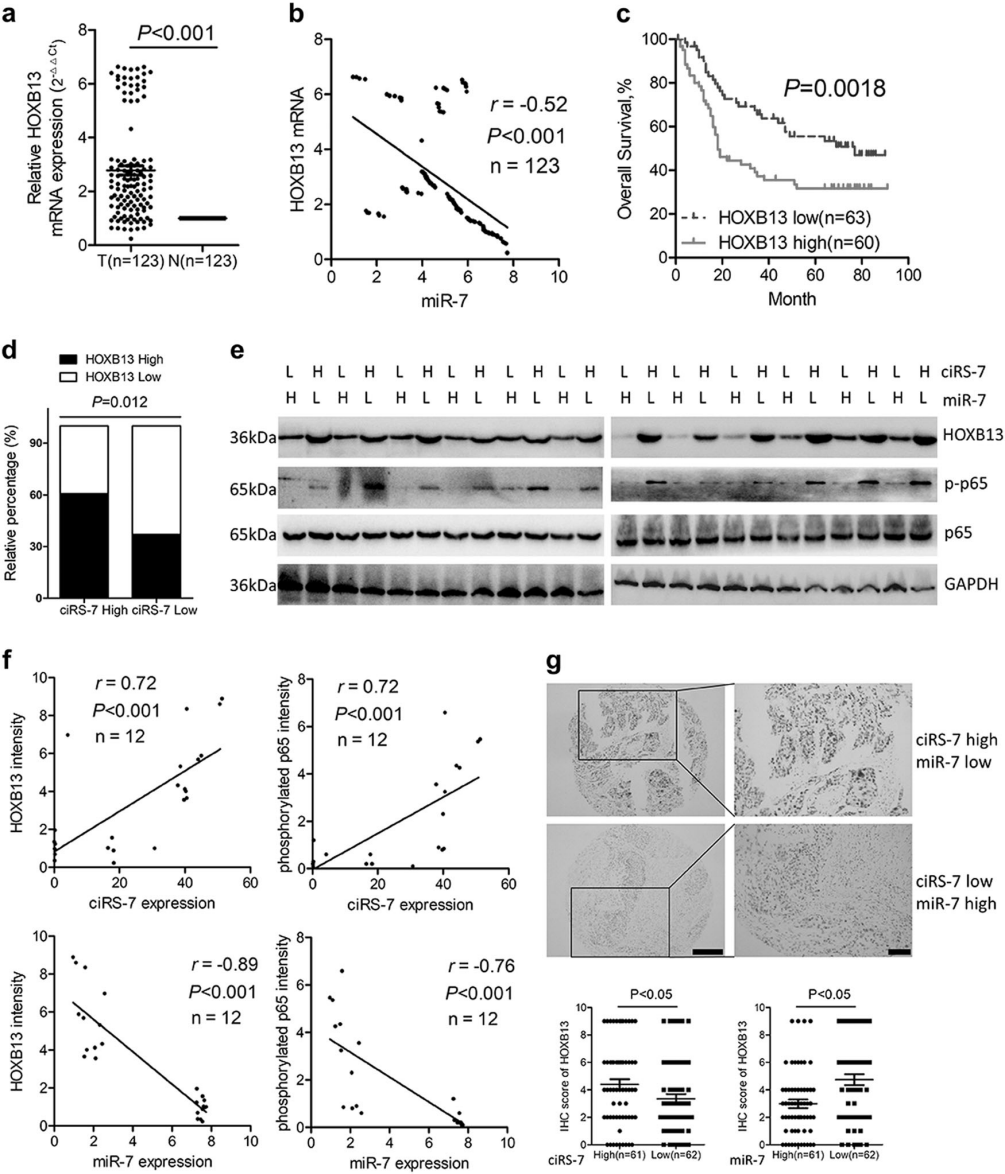
The ciRS-7/miR-7/HOXB13 was aberrantly activated in ESCC. **a** The expression levels of HOXB13 in ESCC tissues and corresponding non-cancerous tissues was detected by qPCR. **b** Correlation between expression level of miR-7 and HOXB13 mRNA in ESCC tumor tissues. **c** Kaplan–Meier analysis of overall survival in ESCC patients with low and high HOXB13 levels based on immunohistochemical scores. **d** Correlations of ciRS-7 and HOXB13 protein expression in ESCC tissues based on immune-scoring. **e** Immunoblots of HOXB13 and p-P65 in 12 paired freshly collected ESCC samples. **f** Significant negative correlations between the levels of miR-7 and indicated proteins and positive correlations between the level of ciRS-7 and indicated proteins were evaluated in ESCC samples. **g** The correlation between the levels of miR-7, ciRS-7, and HOXB13/p-P65 axis in 123 cases of ESCC FFPE tissues. Scale bars: 100 μm. Staining intensities for HOXB13 and p-P65 were significantly reduced in miR-7 high-expressing and ciRS-7 low-expressing ESCC tissues compared with miR-7 low-expressing and ciRS-7 high-expressing tissues. *P < 0.05

**Fig. 7:**
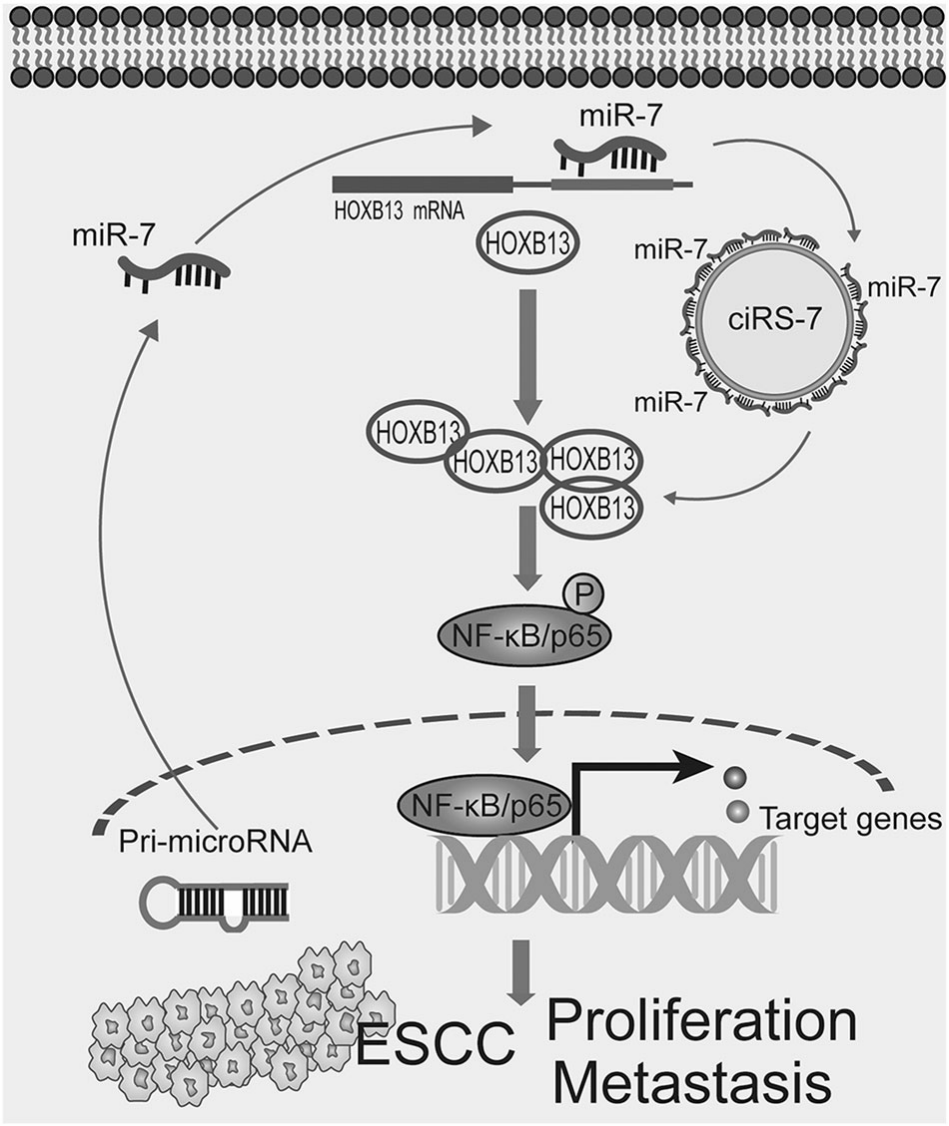
The proposed working model. Upregulation of ciRS-7 promote proliferation and metastasis of ESCC via acting as sponge of miR-7 and reactivating its downstream HOXB13/p-P65 pathway

Expression of HOXB13 was upregulated in ESCC tumor tissues than that in the paired non-cancerous tissues (Fig. 6a) and correlated negatively with miR-7 (Fig. 6b). Based on the staining score of HOXB13, patients were divided into high expression and low expression group. Patients with high HOXB13 expression showed significant shorter overall survival and disease-free survival than those with low HOXB13 expression (Fig. 6c and Supplementary Figure S3A). Expression of HOXB13 was identified as an independent prognosis predictor in ESCC (Table S2). Furthermore, samples with high ciRS-7 or low miR-7 expression tended to show elevated HOXB13 expression (Fig. 6d and Supplementary Figure S3B). Western blot analysis of 12 paired fresh samples further confirmed elevated HOXB13 as well as downstream p65 phosphorylation level in patients with ciRS-7 high and miR-7 low expression than those with ciRS-7 low and miR-7 high expression (Fig. 6e), which was further validated by quantitative analysis (Fig. 6f). Notably, we observed that upregulation of ciRS-7 or downregulation of miR-7 was significantly associated with overexpression of HOXB13 via immunohistochemical analysis (Fig. 6g), highlighting the clinical significance of these results. Altogether, our results suggest that upregulation of ciRS-7 abrogates the tumor-suppressive effect of miR-7 on its downstream targets, HOXB13 and therefore promotes downstream p65 phosphorylation in ESCC (Fig. 7).

## Discussion

In this study, we first discovered that ciRS-7 is significantly upregulated in ESCC tumor tissues and cancer cell lines compared with their normal counterparts. Upregulation of ciRS-7 correlated with several clinical pathological variables and predicted poor outcome of ESCC patients. Second, our data showed that overexpression of ciRS-7 abrogated the tumor-suppressive effects of miR-7 and facilitated malignant progression in ESCC. Third, we unraveled that ciRS-7 reactivated HOXB13 downstream NF-κB/p65 pathways via sponge of miR-7. Conclusively, our results for the first time revealed the prognostic and therapeutic potential of ciRS-7 in this malignancy.

Unlike the linear structure of mature messenger RNA (mRNA) with start and stop termini^34^, circRNA represent a novel class of widespread and diverse endogenous RNAs that regulate gene expression in mammals^17,35^. The circRNAs were first discovered in plants and shown to encode sub viral agents 30 years ago^36^. Until recently, involvement of circRNA in human diseases such as cancer has been reported^15,19,37^. It has been well-accepted that the protein-coding function of mRNAs can be suppressed by the binding of short miRNA sequences and these effects could be abrogated by other non-coding RNAs including lncRNA^38,39^ and circRNA^15,24^, which was designated as the ceRNA mechanism^13^. To function as the ceRNA, complementary sequences between mRNA, miRNA and lncRNA must exit. Previous reports have shown that ciRS-7 contains more than 70 complementary loci for miR-7^16^ and could act as inhibitor, buffer or reservoir of miR-7^17^. Sponge of miR-7 mediated the oncogenic effects of ciRS-7 in gastric^24^ and colorectal cancer^25^. Although we found that ciRS-7 was overexpressed in the ESCC tumor samples and cancer cell lines compared with their normal counterparts, enforced ciRS-7 expression, interestingly, showed no effects on proliferation or migration. By contrast, ciRS-7 could reverse the suppressive roles of miR-7 in ESCC cells, which was consistent with previous reports^24,25^. These data provided solid proof for the therapeutic potential of ciRS-7 inhibition in ESCC.

Recently, several lines of evidence have implicated miR-7 in numerous pathways and diseases^17,40^. For instance, miR-7 has been proposed a role in Parkinson disease via direct regulation of a-synuclein^41^. In addition, miR-7 directly targets and downregulates central oncogenic factors in cancer-associated signaling pathways including EGF receptor^42^, IRS-1^42^, Raf1^22^, PIK3CD^43^, and Kruppel-like factor 4-mediated stem cell formation^44^, indicating a clear tumor-suppressive role for miR-7. Consistent with these reports, overexpression of miR-7 inhibited proliferation of ESCC cells^23,26^. Moreover, the serum miR-7 level was significantly lower in the ESCC patients compared with healthy controls and patients with higher serum miR-7 level tended to benefit from concurrent chemoradiotherapy^45^. Despite overwhelming evidence supporting a tumor-suppressive role of miR-7, the opposite effect has also been reported. For example, overexpression of miR-7 was found to be associated with poor prognosis in lung carcinomas^46^. These apparently contradictory functions of miR-7 may be well explained by the complex downstream genes regulated by miR-7 depending on cancer types^17^. Our data may provide another possible explanation for the paradoxical roles of miR-7 in different cancer as the expression level of ciRS-7 varied according to tumor locations.

HOX genes are essential for mammalian morphogenesis and development^47^. Dysregulation of the HOX gene expression has been shown in diverse cancers^29^. As a member of the HOX gene family, high levels of HOXB13 promote tumorigenesis in different types of tumors such as prostate cancer^33,48^. However, the mechanism underlying HOXB13 overexpression and the roles of HOXB13 in ESCC remain unclear. Here, we provided solid proofs that the post-transcriptional regulation of HOXB13 is partly mediated by ciRS-7 in ESCC progression through competing for miR-7 in cytoplasm. Knockdown of HOXB13 suppressed ESCC cell proliferation and migration. In addition, we found that downstream NF-κB/p65 phosphorylation was reactivated after sponge of miR-7 by ciRS-7 in ESCC cells. Our results showed that overexpression of ciRS-7 may at least in part explain aberrant activation of HOXB13 downstream pathways in ESCC cells besides the lncRNA CCAT1^23^ and supported the hypothesis that ciRS-7 could facilitate malignant progression of ESCC via sponge of miR-7. More importantly, knockdown of ciRS-7 resulted in expansive reactivation and amplification of the tumor-suppressive roles of miR-7 as one molecular ciRS-7 could sponge more than 70 endogenous miR-7 molecular^16^.

Conclusively, our data provide comprehensive evidence that ciRS-7 acts as a novel prognostic biomarker and therapeutic target in ESCC. Upregulation of ciRS-7 impairs tumor-suppressive effects of miR-7 in ESCC cells and in xenografted mouse models. Mechanistically, overexpression of ciRS-7-enhanced HOXB13-mediated NF-κB/p65 phosphorylation through sponge of endogenous miR-7. Strikingly, the ciRS-7/miR-7/HOXB13 axis was aberrantly activated in ESCC and represents a promising therapeutic target in ESCC treatment.

## Materials and methods

### Patient samples

A total of 123 ESCC and corresponding normal adjacent epithelial tissues (NATs, >2 cm from tumor tissues) were obtained postoperatively from patients at the Tongji Hospital from 2009 to 2011, Wuhan, China. There were no restrictions on age, sex, stage of ESCC. Esophageal tissue samples from newly diagnosed ESCC patients were immediately placed in liquid nitrogen and then stored at −80 °C until analysis. All patients provided written consent for use of their tissues with research purpose. None of our cohort received any preoperative treatments. OS was defined as the time from diagnosis to the date of death from any cause or latest follow-up, while DFS was measured from the date of surgery until death, relapse, or second primary tumor, whichever occurred first. This study was approved by the Institutional Review Board of Tongji Medical College of Huazhong University of Science and Technology. Complete clinicopathological parameters and follow-up data for all patients were archived from the medical record. The clinical characteristics are listed in Table 1.

### RNA extraction and real-time quantitative polymerase chain reaction

Total RNA was isolated from cells and tissues using the TRIzol® reagent (Invitrogen) according to the manufacturer’s instructions. Complementary DNA (cDNA) was synthesized from total RNA using SuperScript III® (Invitrogen) according to the supplied protocol. The relative gene expression of ciRS-7 was determined using the LightCycler™ 480 system (Roche Diagnostics) based on the SYBR (QIAGEN) methods, while the small nuclear RNA U48 was used as an internal standard control, and all reactions were performed in triplicate.

For detection of miR-7, cDNA was synthesized from 1 μg total RNA using the miRNA Reverse Transcription kit (Genecopia) according to the manufacturer’s protocol. qPCR was performed in the LightCycler™ 480 system. In brief, 10 μl qPCR mixture included the miRNA-qPCR buffer (Genecopia) and specific primers was incubated in a 384-well optical plate at 95 °C for 10 min, and then subjected to 45 cycles of 95 °C for 10 s, 60 °C for 20 s, and 72 °C for 10 s. Expression levels of miR-7 were normalized to that of U48 and analyzed using the 2^–ΔCt^ methods.

### Cell lines, oligos, and plasmids

Human esophageal cancer cell lines (Eca109, KYSE510, KYSE410, and KYSE150) were obtained either from the Cell Bank of the Chinese Academy of Sciences (Shanghai, China) or purchased from the Deutsche Sammlung von Mikroorganismen und Zellkulturen (DSMZ, Braunschweig, Germany) and cultured at 37 °C with 5% CO_2_ in Dulbecco’s Modified Essential Medium (DMEM) medium (Hyclone, Logan, Utah, USA) supplemented with 10% fetal bovine serum (FBS) (Gibico, Carlsbad, California, USA). Human esophageal epithelial squamous cell NE1 was kindly provided by Dr. Yang XZ at Cancer Hospital and Institute of Guangzhou Medical University (Guangzhou, China) and cultured in a 1:1 mixture of defined keratinocyte serum free medium with growth supplements and EpiLife medium with 60 μM Calcium (Invitrogen, Carlsbad, California, USA).

The miR-7 mimics and ciRS-7 expressing vector were purchased from Genecopia. For transient transfections, Lipofectamine 2000 (Invitrogen, Carlsbad, CA), and Opti-MEM (Gibco, Carlsbad, CA) were used according to the manufacturer’s instructions. Briefly, 20 nM of miR-7 mimics or 2 μg ciRS-7 were used for the functional assays. Lentiviruses with stable ciRS-7 expression or negative controls were constructed as previously reported^25^. For stable transfections, we first established ciRS-7 and negative vector stable expressing Eca109 and KYSE150 cells using G148 (Selleck) selection methods as described previously^25^. The stable cell lines were then infected by miR-7 or negative control virus and selected by puromycin (Selleck) according to the manufacturer’s instructions.

### Cell viability, colony formation, cell migration, and invasion assays

In 96-well, 100 μl of Eca-109 and KYSE150 cell suspensions (500 cells per well) after transfection were aliquoted into each well. Cell viability was measured by the Cell Counting Kit-8 (CCK-8) system (Dojindo) according to the manufacturer’s instructions. Briefly, the plates were incubated at 37 °C for 2 h after addition of 10 μl CCK-8 solution in each well, and the absorbance was read at 490 nm using a microplate reader (BioTek Instruments). There were four replicates for each group, and the experiments were repeated at least three times. Cell cycle analysis was performed as described previously^15^.

For colony formation assays, ESCC cells with stable transfection with ciRS-7, miR-7, and negative controls (500 cells) were seeded into the 6-well plate. After incubation for 10–14 days at 37 °C in a 5% humidified CO_2_ atmosphere, colonies (>50 cells per colony) were stained with crystal violet, counted, and photographed. Each experiment was performed in triplicate. Cell migration and invasion assays were performed using Boyden chambers (Corning, Corning, NY) using 8 μm-size pore membrane coated with matrigel (for invasion assays) or without matrigel (for migration assays) according to previous protocols^49^.

### Western blotting

Protein lysates from ESCC tissues and cells were subjected to western blot analysis according to standard protocols as previously described^50^. Antibody recognizing NF-κB/p65 was from Cell Signaling (CST), while anti-HOXB13 and anti-GAPDH antibodies were from Abcam.

### Luciferase reporter assay

The 3′-UTR of HOXB13 gene containing miR-7-binding sites was amplified and cloned into the pmirGLO Dual-luciferase Expression Vector, termed as pGLS-HOXB13–3′-UTR. Besides, the same putative binding site was mutated and named as pGLS-HOXB13–3′-UTR-Mut. For dual luciferase assays, the cells were transfected with miR-7 mimics or miR-NC, and each group was then transfected with pGLS-HOXB13–3′-UTR or pGLS-HOXB13–3′-UTR-Mut plasmid and pRL-TK reporter gene carrier, respectively, with assistance of Lipofectamine 2000 (Invitrogen, Foster city, CA, USA). The firefly and renilla luciferase activities were measured by applying dual luciferase reporter assay system (Promega) in 48 h after transfection according to the manufacturer’s instructions. All the assays were repeated for at least 34 times.

### Immunohistochemical analysis

For IHC staining, the formalin-fixed paraffin embedded ESCC tissues of abovementioned cases were collected. IHC analyses were conducted according to the standard procedures. Briefly, the slides were blocked and incubated with anti-HOXB13 (1:200 dilution) antibodies at 4 °C overnight. Tissue sections were then incubated with biotinylated goat anti-rabbit immunoglobulin at 37 °C for 30 min. Finally, the sections were stained with diaminobenzidine (DAB). Two independent observers who were blinded to the clinical characteristics and outcomes of the patients assessed the IHC staining based on the proportion and intensity of positively stained tumor cells. For each tissue, the proportion of HOXB13-positive cells varied from 0 to 100% (scores of the positive staining proportion: 1, <25%; 2, 25–50%; 3, 50–75%; 4, 75–100%), and the staining intensity varied from negative to strong. A final score was calculated by multiplying these two parameters. The HOXB13 expression level was considered high when the final scores were ≥ median (score = 4) and low when the final scores were < median (score = 4).

### Animal study

All BALB/c nude mice (4 weeks old, female) were maintained under pathogen free conditions and all procedures for the mouse experiments were approved by the Animal Care Committee of Tongji Medical College. Eca109/NC, ciRS-7, miR-7, or ciRS-7/miR-7 cells (1 × 10^6^ cells/mouse) were subcutaneously injected into the right flank of BALB/C nude mice (*n* = 6/group). The tumor volumes were monitored every 3 days after injection. All mice were sacrificed 3 weeks afterwards, and the xenografts were dissected out for qPCR or immunohistochemical analysis. For the metastasis model, the abovementioned cells (2 × 10^6^ cells/mouse) were injected into the tail vein of nude mice (*n* = 8/group). Six weeks post injection, the mice were killed, and the lungs were removed, and paraffin embedded. Consecutive sections (4 μm) were made and stained with hematoxylin–eosin. The micro metastases in the lungs were examined and counted.

### Statistical analysis

Each experiment was repeated three or more times. Unless otherwise noted, data are presented as the mean ± S.D., and Student’s *t*-tests (unpaired, two-tailed) were conducted using Prism GraphPad software (San Diego, CA, USA). Survival curves were generated using the Kaplan–Meier method and assessed with the log-rank tests using statistical software (SPSS 19.0 for Windows; SPSS Inc., Chicago, IL). The Cox proportional hazard regression model was used to identify independent prognostic factors. A *P* < 0.05 was considered statistically significant.

## Electronic supplementary material

supplementary table 1

supplementary table 2

supplementary figure 1

supplementary figure 2

supplementary figure 3

supplementary figure legends
