# Supplementary material for: CiRS-7 promotes growth and metastasis of esophageal squamous cell carcinoma via regulation of miR-7/HOXB13
Source: Cell Death Dis. 2018 Aug 6;9(8):838. doi: 10.1038/s41419-018-0852-y (PMC6079012; doi:10.1038/s41419-018-0852-y)
Supplement: Supplementary file 1 — supplementary table 1 [file 41419_2018_852_MOESM1_ESM.docx]

**Supplementary Table 1.** The correlation between clinicopathological parameters and miR-7 expression

|  | miR-7 expression | | *P* |
| --- | --- | --- | --- |
|  | Low, n (%) | High, n (%) |  |
| Age |  |  |  |
| ≤ 50 | 7(30.4) | 16(69.6) | 0.039* |
| > 50 | 55(55.0) | 45(45.0) |  |
| Gender |  |  |  |
| Male | 51(51.5) | 48(48.5) | 0.655 |
| Female | 11(45.8) | 13(54.2) |  |
| Alcohol consumption |  |  |  |
| Ever and current | 44(53.0) | 39(47.0) | 0.445 |
| Never | 18(45.0) | 22(55.0) |  |
| Smoking status |  |  |  |
| Ever and current | 27(49.1) | 28(50.9) | 0.857 |
| Never | 35(51.5) | 33(48.5) |  |
| Tumor size |  |  |  |
| < 5cm | 49(47.6) | 54(52.4) | 0.222 |
| ≥ 5cm | 13(65.0) | 7(35.0) |  |
| Differentiation status |  |  |  |
| Well or Moderate | 46(48.4) | 49(51.6) | 0.520 |
| Poor | 16(57.1) | 12(42.9) |  |
| TNM stage |  |  | 0.281 |
| I-II | 28(45.2) | 34(54.8) |  |
| III | 34(55.7) | 27(44.3) |  |
