# Supplementary material for: CiRS-7 promotes growth and metastasis of esophageal squamous cell carcinoma via regulation of miR-7/HOXB13
Source: Cell Death Dis. 2018 Aug 6;9(8):838. doi: 10.1038/s41419-018-0852-y (PMC6079012; doi:10.1038/s41419-018-0852-y)
Supplement: Supplementary file 2 — supplementary table 2 [file 41419_2018_852_MOESM2_ESM.docx]

**Supplementary Table 2.** Univariate and multivariate analyses of various potential prognostic factors in ESCC patients

|  | Univariate analysis | | Multivariate analysis | |
| --- | --- | --- | --- | --- |
|  | HR (95% CI) | *P* | HR (95% CI) | *P* |
| Age (≤50/>50) | 1.14(0.61-2.12) | 0.690 | - | - |
| Gender (Male/Female) | 1.03(0.57-1.86) | 0.918 | - | - |
| Alcohol (Never/Ever) | 1.00(0.60-1.64) | 0.988 | - | - |
| Smoke (Never/Ever) | 0.85(0.53-1.37) | 0.506 | - | - |
| Tumor size  (≥5cm/<5cm) | 1.41(0.76-2.63) | 0.282 | - | - |
| Differentiation  (Poor/Moderate, Well) | 1.26(0.74-2.16) | 0.399 | - | - |
| TNM Stage(III/I-II) | 3.14(1.90-5.20) | 0.000* | 3.31(1.99-5.51) | 0.000* |
| HOXB13 (High/Low) | 1.84(1.14-2.97) | 0.013* | 1.99(1.23-3.23) | 0.005* |

HR: hazard ratio; CI: confidence interval; **P* < 0.05.
