# Supplementary figures and images for: CiRS-7 promotes growth and metastasis of esophageal squamous cell carcinoma via regulation of miR-7/HOXB13
Source: Cell Death Dis. 2018 Aug 6;9(8):838. doi: 10.1038/s41419-018-0852-y (PMC6079012; doi:10.1038/s41419-018-0852-y)

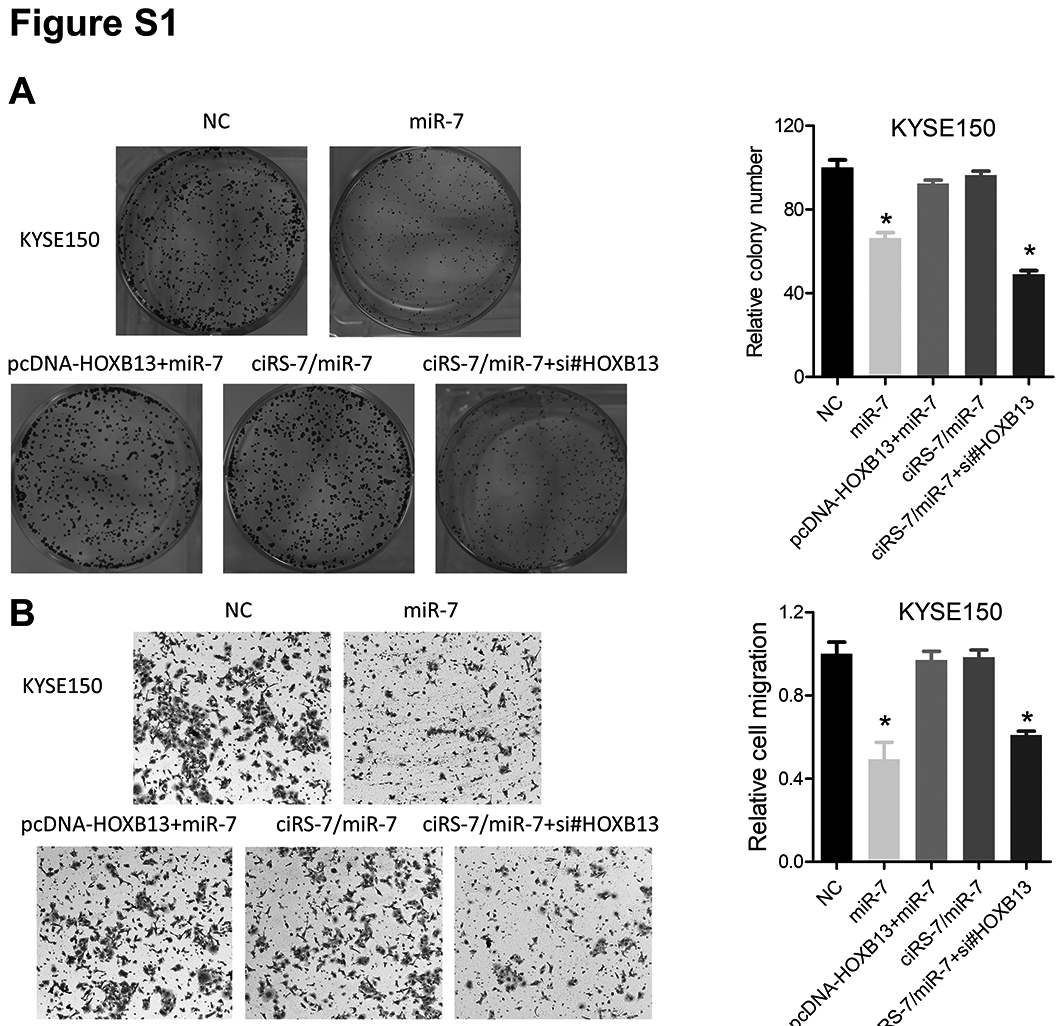

Supplement: Supplementary file 3 — supplementary figure 1 [file 41419_2018_852_MOESM3_ESM.tif]

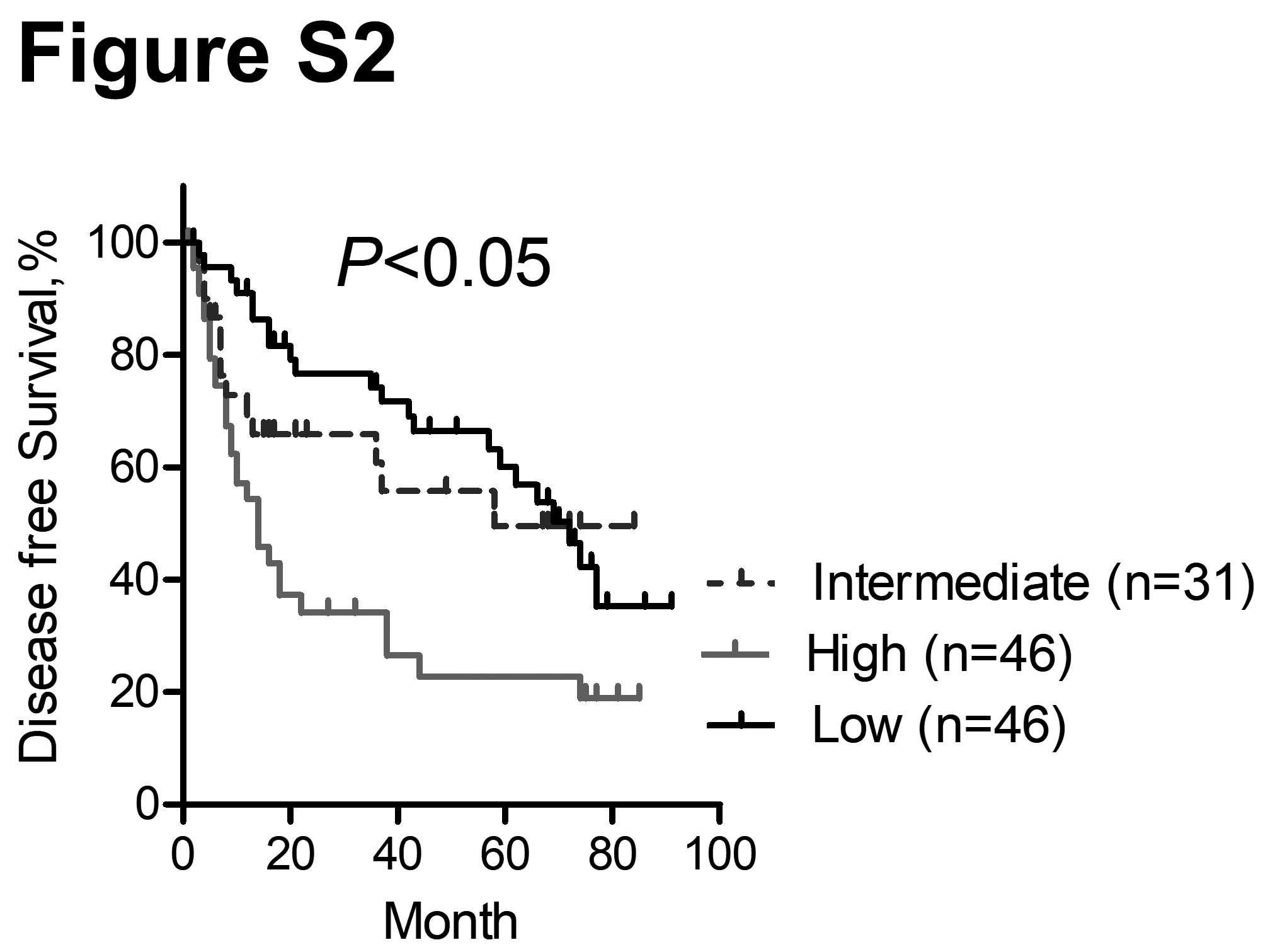

Supplement: Supplementary file 4 — supplementary figure 2 [file 41419_2018_852_MOESM4_ESM.tif]

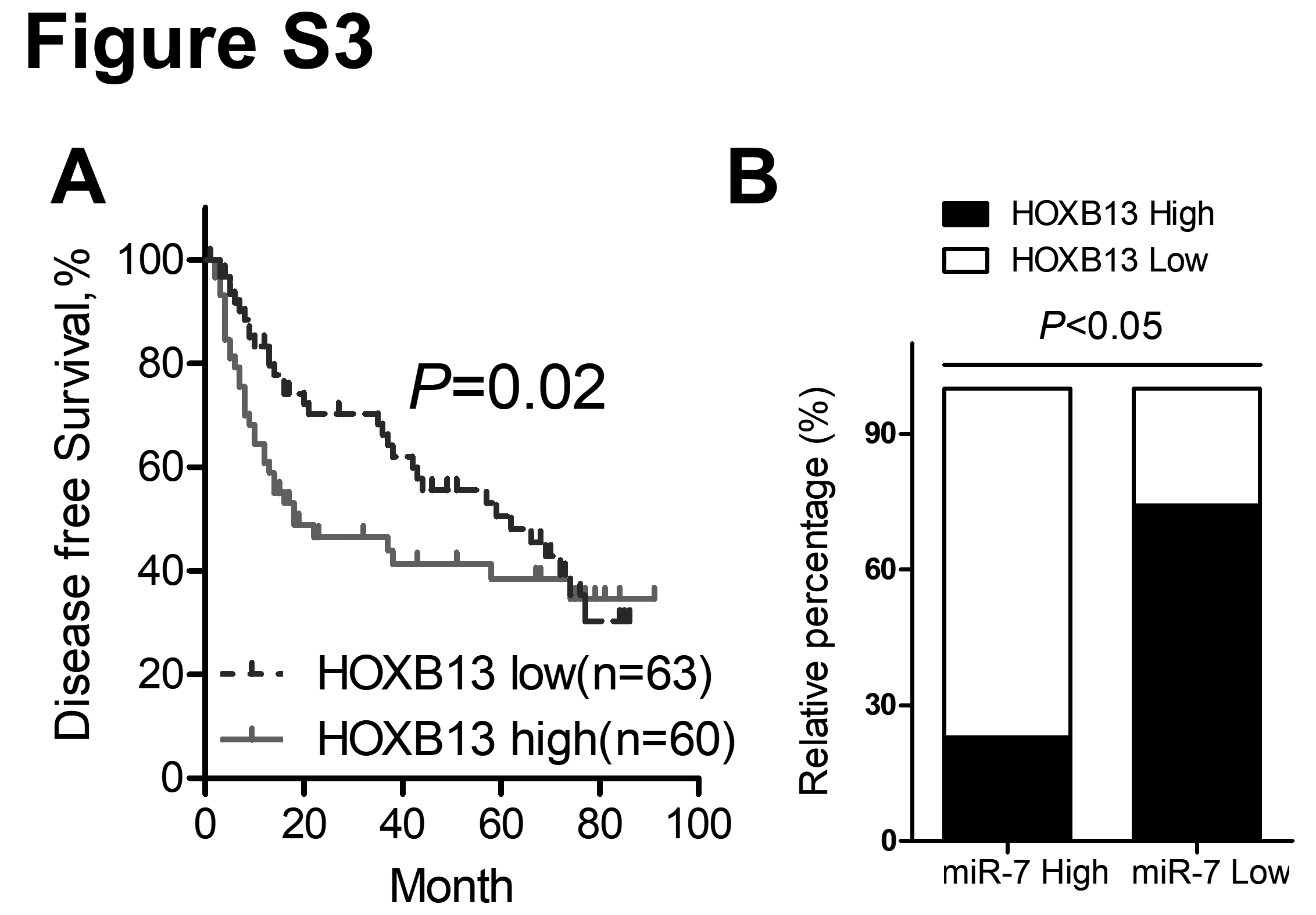

Supplement: Supplementary file 5 — supplementary figure 3 [file 41419_2018_852_MOESM5_ESM.tif]
