# Supplementary material for: CiRS-7 promotes growth and metastasis of esophageal squamous cell carcinoma via regulation of miR-7/HOXB13
Source: Cell Death Dis. 2018 Aug 6;9(8):838. doi: 10.1038/s41419-018-0852-y (PMC6079012; doi:10.1038/s41419-018-0852-y)
Supplement: Supplementary file 6 — supplementary figure legends [file 41419_2018_852_MOESM6_ESM.docx]

**Supplementary figure 1. The ciRS-7/miR-7 promotes proliferation and migration of ESCC cells via HOXB13.** (A) Colony formation assays of KYSE150 cells after overexpression of ciRS-7, miR-7, HOXB13 or siRNA transfection. Left panel was representative images and right panel was statistical quantification. (B) Migration assays of KYSE150 cells after overexpression of ciRS-7, miR-7, HOXB13 or siRNA transfection. Left panel was representative images and right panel was statistical quantification. Data in A and B represents the mean ± SD of three repeated experiments. **P* < 0.05.

**Supplementary figure 2. The ciRS-7/miR-7 correlates with poor prognosis in ESCC.** Disease-free survival of ESCC patients based on expression of ciRS-7 and miR-7. **P* < 0.05.

**Supplementary figure 3. The ciRS-7/miR-7/HOXB13 was aberrantly activated in ESCC.** (A) Kaplan–Meier analysis of disease-free survival in ESCC patients with low and high HOXB13 levels based on immunohistochemical scores. (B) Correlations of miR-7 and HOXB13 protein expression in ESCC tissues based on immune-scoring. **P* < 0.05.
